# Defining terms commonly used in sarcopenia research: a glossary proposed by the Global Leadership in Sarcopenia (GLIS) Steering Committee

**DOI:** 10.1007/s41999-022-00706-5

**Published:** 2022-11-29

**Authors:** Peggy M. Cawthon, Marjolein Visser, Hidenori Arai, José A. Ávila-Funes, Rocco Barazzoni, Shalender Bhasin, Ellen Binder, Olivier Bruyère, Tommy Cederholm, Liang-Kung Chen, Cyrus Cooper, Gustavo Duque, Roger A. Fielding, Jack Guralnik, Douglas P. Kiel, Ben Kirk, Francesco Landi, Avan A. Sayer, Stephan Von Haehling, Jean Woo, Alfonso J. Cruz-Jentoft

**Affiliations:** 1 California Pacific Medical Center, Research Institute, 550 16th Street, Second Floor, San Francisco, CA 94143 USA; 2 Department of Epidemiology and Biostatistics, University of California San Francisco, San Francisco, CA USA; 3 Department of Health Sciences, Faculty of Science, Vrije Universiteit Amsterdam, Amsterdam, The Netherlands; 4 The Amsterdam Public Health Research Institute, Amsterdam, The Netherlands; 5 National Center for Geriatrics and Gerontology, Obu, Aichi Japan; 6 Department of Geriatrics, Instituto Nacional de Ciencias Médicas y Nutrición Salvador Zubirán, Mexico City, Mexico; 7 Department of Medical, Surgical and Health Sciences, University of Trieste, Trieste, Italy; 8 Bostin Claude D. Pepper Older Americans Independence Center, Brigham and Women’s Hospital, Harvard Medical School, Boston, MA USA; 9 Siteman Cancer Center at Barnes-Jewish Hospital, St. Louis, MO USA; 10 Division of Geriatrics and Nutritional Science, Washington University School of Medicine, St. Louis, MO USA; 11 Division Public Health, Epidemiology and Health Economics, World Health Organization Collaborating Centre for Public Health Aspects of Musculoskeletal Health and Aging, University of Liège, Liège, Belgium; 12 Department of Public Health and Caring Sciences, Clinical Nutrition and Metabolism, Uppsala University, Uppsala, Sweden; 13 Theme Inflammation and Ageing, Karolinska University Hospital, Stockholm, Sweden; 14 Center for Healthy Longevity and Aging Sciences, National Yang Ming Chiao Tung University, Taipei, Taiwan; 15 Center for Geriatrics and Gerontology, Taipei Veterans Generfranal Hospital, Taipei, Taiwan; 16 Taipei Municipal Gan-Dau Hospital (Managed by Taipei Veterans General Hospital), Taipei, Taiwan; 17 MRC Lifecourse Epidemiology Unit, University of Southampton, Southampton, UK; 18 Department of Epidemiology, University of Oxford, Oxford, OX UK; 19 Research Institute of the McGill University Health Centre, Montreal, QC Canada; 20 Division of Geriatric Medicine, Department of Medicine, McGill University, Montreal, QC Canada; 21 Nutrition Exercise, Physiology, and Sarcopenia Laboratory, Jean Mayer U.S. Department of Agriculture Human Nutrition Research Center On Aging, Tufts University, Boston, MA USA; 22 Department of Epidemiology and Public Health, University of Maryland School of Medicine, Baltimore, MD USA; 23 Hinda and Arthur Marcus Institute for Aging Research, Hebrew SeniorLife, Department of Medicine Beth Israel Deaconess Medical Center and Harvard Medical School, Boston, MA USA; 24 Australian Institute for Musculoskeletal Science (AIMSS), The University of Melbourne and Western Health, St. Albans, VIC Australia; 25 Department of Medicine-Western Health, The University of Melbourne, St. Albans, VIC Australia; 26 Fondazione Policlinico Universitario “Agostino Gemelli” IRCCS, 00168 Rome, Italy; 27 AGE Research Group, NIHR Newcastle Biomedical Research Centre, Newcastle Upon Tyne Hospitals NHS Foundation Trust and Faculty of Medical Sciences, Newcastle University, Newcastle, UK; 28 Department of Cardiology and Pneumology, University Medicine Göttingen (UMG), Göttingen, Germany; 29 German Centre for Cardiovascular Research (DZHK), Partner Site Göttingen, Göttingen, Germany; 30 Department of Medicine and Therapeutics, Faculty of Medicine, The Chinese University of Hong Kong, Hong Kong, China; 31 Faculty of Medicine, Centre for Nutritional Studies, The Chinese University of Hong Kong, Hong Kong, China; 32 Servicio de Geriatría, Hospital Universitario Ramón y Cajal (IRYCIS), Madrid, Spain

**Keywords:** Sarcopenia, Terminology, Standardization, GLIS

## Abstract

**Methods:**

The aim of this paper is to define terms commonly related to sarcopenia to enable standardization of these terms in research and clinical settings. The Global Leadership Initiative in Sarcopenia (GLIS) aims to bring together leading investigators in sarcopenia research to develop a single definition that can be utilized worldwide; work on a global definition of sarcopenia is ongoing. The first step of GLIS is to develop the common terminology, or a glossary, that will facilitate agreement on a global definition of sarcopenia as well as interpretation of clinical and research findings.

**Results:**

Several terms that are commonly used in sarcopenia research are defined, including self-reported measures of function and ability; objective physical performance tests; and measures related to muscle function and size.

**Conclusion:**

As new methods and technologies are developed, these definitions may be expanded or refined over time. Our goal is to promote this common language to describe sarcopenia and its components in clinical and research settings in order to increase clinical awareness and research interest in this important condition. We hope that the use of common terminology in sarcopenia research will increase understanding of the concept and improve communication around this important age-related condition.

## Key summary points

**Aim:**

The aim of this paper is to define terms commonly related to sarcopenia to enable standardization of these terms in research and clinical settings.

**Findings:**

This paper provides definitions for commonly used terminology in sarcopenia in both clinical and research settings. As new methods and technologies are developed, this terminology may be expanded or refined over time.

**Message:**

We hope that the use of common terminology in sarcopenia research will increase understanding of the concept and improve communication around this important age-related condition.

## Introduction

The aim of this paper is to define terms commonly related to sarcopenia to enable standardization of these terms in research and clinical settings. The Global Leadership Initiative in Sarcopenia (GLIS) aims to bring together leading investigators in sarcopenia research to develop a single definition of sarcopenia that can be utilized worldwide. The first step of GLIS is to develop the common terminology, or a glossary, that will facilitate future agreement on a global definition of sarcopenia as well as interpretation of clinical and research findings. Researchers and clinicians must first use a common set of terms related to sarcopenia before there can be global agreement on a definition of sarcopenia; this paper reflects the effort to speak a common language to facilitate the future development of a global sarcopenia definition. The future work of GLIS will be to develop a global definition of sarcopenia using a modified Delphi approach. This paper will establish that common language.

There have been many proposed definitions of sarcopenia that vary in terms of the components included and the description of these components [1–7]. The definitions also vary with respect to specific cut-points used to define each component. For example, the International Working group uses a cut-point in gait speed of < 1.0 m/s to define slowness, while the European Working Group for Sarcopenia in Older People II approach uses a cut-point in gait speed of ≤ 0.8 m/s to define poor physical performance. The proposed definitions also differ in terminology in how the components of the sarcopenia definitions are described. The goal of this paper is to standardize language around the components of these definitions, and other factors related to sarcopenia. Recommendations for specific components to include in a global definition of sarcopenia (and potentially cut-points for these components) will be the subject of future work. Further, this paper will not review screening tools for sarcopenia case finding (e.g., SARC-F) [8], as such a review is outside the scope of our goal to define commonly used terms.

This paper is organized by terminology for general domains of (1) *self-reported function*; (2) objective measures of integrative *physical performance* that includes, for example, walking performance and repeat chair stands*;* (3) *muscle function* that includes strength and specific force; and (4) *muscle quantity* and other muscle metrics, which includes measures of “muscle quantity” and other factors related to quantitative muscle traits (radiodensity from computed tomography (CT), etc.)

We acknowledge that while sarcopenia is related to disability, it is a distinct entity from disability, and there have been several different frameworks have been proposed to describe the process of disability [9–13]. Depending on the framework used, functional limitations, impairments, and disability are important components or consequences of sarcopenia. The focus of this report is to clarify terms related to sarcopenia; we will refrain from defining these terms within the context of a specific disability framework. We note, however, that “impairment”, “limitation”, “difficulty”, and “disability” are often used interchangeably in research reports without an explicit description of how these measures are assessed (e.g., by objective assessment of performance or by self-report) or what disability framework is used. When such terms are used, it is recommended that the disability framework used to define these terms be cited and the assessment of the metric under study be explicitly described.

The specific terminology proposed is outlined below.

### Self-reported measures of function

Self-reported measures of function and ability generally indicate the person’s capacity in their own social and physical context in which functioning actually takes place [14]. These measures indicate the perceived ability or the perceived difficulty to complete activities of daily living (ADLs), instrumental activities of daily living (IADLs) and mobility activities. Many different tasks can be assessed, including eating, bathing, transferring, walking short and long distances, handling day-to-day management of life tasks (money, medications), etc. Scales have been developed that include a range of (I)ADL tasks, such as the Barthel Index for Activities of Daily Living [15]. When reporting these tasks, the language should be clear that these measures were based on self-report (or reported by proxy contact if appropriate).

### Objective physical performance tests

A physical performance measure tests the capacity of a person in a standardized setting. Performance tests often have greater sensitivity than self-reported function in the higher ability range, so that they can detect functional decline that is still imperceptible to respondents and thus not identified by self-reports [16, 17]. There are numerous tests to objectively assess physical performance, including walking tests (over short and long distances, from less than 4–400 m or more); balance tests; the repeated chair stands test; the timed-up and go test (TUG); the stair climb test [18]; the four-square test [19]; and others. The Short Physical Performance Battery (SPPB) is a frequently used set of three performance tests, including a 3- or 4-m walk test, repeated chair stands test, and a balance test [20]. The time needed to perform the test or a set of tests, in seconds, is used as an indicator of physical performance and summarized as a score (range 0–12 with higher scores indicating better performance). Inability to complete the 400 m walk within 15 min has been used as a measure of objective mobility disability [21, 22], although some have argued this is more a measure of functional capacity (as it is timed performance) than disability (as it is not a necessary activity to carry out in ones’ daily life).

### Muscle function

Muscle function is the objectively measured assessment of integrative muscle function and includes measures of strength and power. Specifically, this includes:

*Muscle strength* This is a measure of strength (or force) generated by a muscle, often expressed in Newton (N) or kilogram (kg). Often the maximal strength generating capacity is measured. In sarcopenia research as well as in clinical practice, grip strength is commonly assessed. Another measure of muscle strength is leg extension strength. Strength may be measured isometrically (contraction without shortening of the muscle), isotonically (contraction with shortening without fixing velocity), or isokinetically (contraction with shortening at fixed velocity).

*Muscle power* This is a measure combining both force and how quickly the force can be generated, expressed in Watts (W). This can be measured by several approaches including leg press machines [23], jumping mechanography [24], or jump height [25]; power can also be estimated from a validated equation using sit-to-stand time.

*Muscle specific force* Specific force (or specific strength) is defined as strength standardized to muscle size (e.g. leg extension 1RM (repetition max) standardized to quadriceps volume or computed tomography (CT) cross-sectional area or leg soft tissue lean mass). This is also sometimes referred to as “muscle quality” but as noted below, the term “muscle quality” should be avoided as it is imprecise. Specific force can also refer to in vitro force production/fiber cross-sectional area in single muscle fibers obtained from biopsy. The units of muscle specific force measures are the ratio of strength to muscle quantity and depend on the exact measures used, examples include Newton/cm^2^, Newton/kg, etc.

### Muscle quantity

Many different approaches to approximate the amount of muscle mass are used in humans. When describing measures of muscle mass or size, it is important to consistently and precisely describe the metric and method used for the approximation (e.g., “DXA lean mass” or “CT CSA”). Below are several commonly used terms for the approximation of muscle mass.

*Lean mass* or soft-tissue lean mass, is the non-bone/non-fat component of the body that includes muscle mass, connective tissue, body organs, water and other materials. This is measured by dual energy x-ray absorptiometry (DXA, “*DXA whole-body lean mass*”), Often, DXA appendicular lean mass (“*DXA ALM*”), or the sum of the soft-tissue lean mass of the arms and the legs, is analysed, with or without adjustment for body size (e.g., DXA ALM/ht^2^) [26]. Despite being an estimate of muscle mass, lean mass has been historically used as a surrogate for muscle mass in sarcopenia research. ALM can be predicted using bioimpedance analysis (“*BIA whole body lean mass*”).

*Fat-free mass (FFM)* is the non-fat component of the body that includes both lean mass and bone mass. This is measured by DXA, underwater-weighing, deuterium dilution, or air displacement plethysmography (BodPod), and can be predicted using BIA.

*Muscle cross-sectional area (CSA) or muscle volume* These measures are derived from computed tomography (CT), magnetic resonance imaging (MR), or anthropometry. Muscle CSA from CT (*CT muscle CSA*) is a commonly used metric to define sarcopenia in several specialty areas where routine CT scans are completed (oncology, GI disease, etc.); in research settings, mid-thigh CSA is often the metric employed [27]. While less commonly used, muscle volume can also be determined from serial CT scans or serial MR scans (e.g., MR muscle volume) [28]. It is somewhat less common due to previously arduous requirements for manual segmentation of body compartments, but newer automated techniques, are making these measures more widely available, at least in research settings. Using a combination of the anthropometric measurements such as circumference and skinfolds at the mid-upper arm or calf, a rough estimation of muscle cross-sectional area can be obtained; however, this method can suffer from relatively large intra- and inter-examiner measurement error.

*D*_*3*_*Cr muscle mass* uses a timed isotope dilution approach (includes participant dosing and a urine sample collection) to estimate creatine pool size, and then muscle mass (kg). It is often analyzed standardized to body size (D_3_Cr muscle mass/weight).

*Ultrasound assessment of muscle size* Ultrasound technology can be employed at the bedside to measure, at standardized anatomical landmarks, parameters that may allow for the estimation of muscle thickness and cross-sectional area [29, 30].

### Other muscle metrics

There are a number of other muscle characteristics aside from muscle quantity that are described in the field of sarcopenia.

*Muscle quality* This is a general term that broadly describes qualities of muscle beyond mass that can include histological, imaging, metabolic, or functional/impairment assessments. However, this term should be avoided when describing specific variables in an analysis or results because it may have several competing specific meanings (see below).

*Muscle density* Muscle density, also known as radiodensity or muscle attenuation from CT, is a measure of the attenuation of X-rays through the muscle tissue and is expressed in Hounsfield units (HU). Muscle density can be assessed on many CT imaging modalities (central CT, high resolution peripheral quantitative computed tomography, HR-pQCT). Radiodensity can only be measured by imaging methods that employ radiation; thus muscle density cannot be measured by magnetic resonance imaging. Higher muscle density has been interpreted as muscle tissue with lower fat content [31]. Muscle density is sometimes referred to as “muscle quality”, but this term should be avoided.

*Muscle texture* This refers to metrics that quantify the “texture” of muscle on an image, usually CT (but other imaging modalities are possible), that measure the spatial arrangement, contrast, and consistency of the greyscale pixels in the image.

*Myosteatosis* This is considered a marker of fat infiltration into muscle. It has been used interchangeably for intra-muscular fat infiltration and intermuscular fat infiltration. Thus, the term “myosteatosis” is non-specific when reporting analysis results; specific terms should be used instead rather than the general term myosteasotosis.

*Muscle fat infiltration* There are numerous terms that have been used to describe fat, adipose tissue, lipids, and/or triglycerides found near or within the muscle fascia. These include intramuscular adipose tissue, intermuscular adipose tissue, intramyocellular lipids (IMCL), and extramyocellular lipids (EMCL). These terms, at times, have conflicting definitions in the literature, and these factors can be measured by various methodologies depending on the precise facet under study, including magnetic resonance imaging or spectroscopy, computed tomography, and analysis of muscle tissue through histologic staining, or other approaches. One of the most common assessments of muscle fat infiltration in aging research is **CT muscle density** (or radiodensity) which is measure of the attenuation of the X-ray through the muscle tissue and expressed in Hounsfield units (HU). CT muscle density is moderately to strongly correlated with muscle biopsy measures of triglycerides and lipids [31]. As the focus of this paper is sarcopenia per se (and not sarcopenic obesity), we refrain from making specific recommendations about terminology about muscle fat at this time, but note that precision of language is of particular importance here: investigators should strive to avoid general terms (e.g., myostaeatosis) when describing specific results.

*Ultrasound based measures* Ultrasound can also be used at the bedside to measure other anatomical characteristics of muscles (pennation angle, fascicle length and echo-intensity) [29, 30].

## Discussion

This paper provides definitions for commonly used terminology in sarcopenia in both clinical and research settings. As new methods and technologies are developed, these definitions may be expanded or refined over time.

The statements above require some additional considerations. First, measures of muscle quantity are often standardized for body size; the analysis variable is therefore a ratio (e.g., ALM/ht^2^). The use of ratios in statistical models is controversial. This is because in some circumstances the use of ratios can lead to spurious associations; and due to concerns that adjustment for the standardization factor (i.e., height) in a multivariate model can be problematic for interpretation of the effect estimates. An alternative is to adjust multivariate models for the body size standardization variable.

Our goal is to promote this common language to describe sarcopenia and its components in clinical and research settings in order to increase clinical awareness and research interest in this condition. We hope that the use of common terminology in sarcopenia research will increase understanding of the concept and improve communication around this age-related condition.
